# Whole-Body Cryotherapy Increases the Activity of Nitric Oxide Synthase in Older Men

**DOI:** 10.3390/biom11071041

**Published:** 2021-07-16

**Authors:** Magdalena Wiecek, Zbigniew Szygula, Joanna Gradek, Justyna Kusmierczyk, Jadwiga Szymura

**Affiliations:** 1 Department of Physiology and Biochemistry, Institute of Biomedical Sciences, Faculty of Physical Education and Sport, University of Physical Education in Kraków, 31-571 Kraków, Poland; justyna.kusmierczyk@awf.krakow.pl; 2 Department of Sports Medicine and Human Nutrition, Institute of Biomedical Sciences, Faculty of Physical Education and Sport, University of Physical Education in Kraków, 31-571 Kraków, Poland; zbigniew.szygula@awf.krakow.pl; 3 Department of Athletics, Faculty of Physical Education and Sport, University of Physical Education in Kraków, 31-571 Kraków, Poland; joanna.gradek@awf.krakow.pl; 4 Department of Clinical Rehabilitation, Faculty of Motor Rehabilitation, University of Physical Education in Kraków, 31-571 Kraków, Poland

**Keywords:** whole-body cryostimulation, aging, oxidative stress, nitric oxide synthase, vasodilatation, endurance athletes

## Abstract

Aging causes oxidative stress, endothelial dysfunction and a reduction in the bioavailability of nitric oxide. The study aim was to determine whether, as a result of repeated whole-body exposure to cryogenic temperature (3 min −130 °C), there is an increase of inducible nitric oxide synthase (iNOS) concentration in senior subjects (59 ± 6 years), and if this effect is stronger in athletes. In 10 long-distance runners (RUN) and 10 untraining (UTR) men, 24 whole-body cryotherapy (WBC) procedures were performed. Prior to WBC, after 12th and 24th treatments and 7 days later, the concentration of iNOS, asymmetric dimethylarginine (ADMA), 3-nitrotyrosine (3-NTR), homocysteine (HCY), C-reactive protein (CRP) and interleukins such as: IL-6, IL-1β, IL-10 were measured. In the RUN and UTR groups, after 24 WBC, iNOS concentration was found to be comparable and significantly higher (F = 5.95, *p* < 0.01) (large clinical effect size) compared to before 1st WBC and after 12th WBC sessions. There were no changes in the concentration of the remaining markers as a result of WBC (*p* > 0.05). As a result of applying 24 WBC treatments, using the every-other-day model, iNOS concentration increased in the group of older men, regardless of their physical activity level. Along with this increase, there were no changes in nitro-oxidative stress or inflammation marker levels.

## 1. Introduction

The main vasodilatory mediator secreted by endothelial cells is nitric oxide (NO). NO also inhibits the expression of proinflammatory cytokines, leukocyte adhesion factors, reduces the adhesion and aggregation of thrombocytes, and has anti-atherosclerotic effects, that is why endothelium has an endocrine effect influencing systemic homeostasis [1,2].

NO is formed from L-arginine in a reaction catalysed by nitric oxide synthase isoforms (NOS) [3]. The bioavailability of NO is influenced by regular physical activity, which also shows an antioxidant effect [4]. During muscle cell contractions, NO synthesis rises as a result of increased activity of endothelial nitric oxide synthase (eNOS), also located in the sarcoplasm, muscle mitochondria and sarcolemma. NO is additionally produced as a result of neuronal (nNOS) and inducible (iNOS) nitric oxide synthase activation. nNOS and iNOS are located in neurons, myocytes, as well as monocytes and macrophages [5,6,7].

The endogenous NOS inhibitor is asymmetric dimethylarginine (ADMA), the elevated level of which is a risk factor for cardiovascular disease (CVD) and atherosclerosis [8,9,10]. There is also a significant relationship between hyperhomocysteinemia and CVD, as well as complications such as myocardial infarction and stroke [11]. The atherogenic effect of homocysteine (HCY) may result from its influence on the reduction in high-density lipoprotein (HDL) concentration, disturbances in fibrinolysis, oxidative stress intensification as well as reduction in the bioavailability of NO, thereby increasing vasoconstriction. Homocysteine reacts with NO to form S-nitrohomocysteine, decreasing the concentration of NO. It is also known that, as a result of endothelial cell exposure to HCY or its precursor—L-methionine, the concentration of ADMA in a cell-culture medium increases significantly. It has been proved that HCY post-translationally inhibits the activity of DDAH (dimethylarginine dimethylamino hydrolase), an enzyme that degrades ADMA, causing the accumulation of ADMA and inhibition of NO synthesis [12]. HCY concentration increases with age and its level is higher in men than in women [13].

However, the excessive production of NO, as a result of iNOS activation, may be disadvantageous, because along with a high level of the superoxide radical anion (O_2_^•^^−^), a highly reactive peroxynitrite (OONO^−^) is produced while reducing the bioavailability of NO. The consequence of nitro-oxidative stress is cell damage at a molecular level, uncontrolled change in the permeability of biological membranes and dysfunction of enzymatic proteins [14]. Nitro-oxidative stress is one of the factors causing disorders related to endothelial cell dysfunction, such as: dyslipidemia, atherosclerosis, CVD and inflammatory diseases, metabolic syndrome and type-2 diabetes [15,16].

Currently, more and more scientific research is concerned with the possibility of using whole-body cryotherapy (WBC) as a method having beneficial effects on the body, *inter alia*, by influencing the processes of lipid and glucose metabolism, having anti-inflammatory effects, and increasing the body’s antioxidant capacity [17,18,19,20].

During WBC procedures, consisting in short-term, often repeated exposure to cryogenic temperatures (1–3 min, 5–30 treatments, temperature from −100 °C to −160 °C) [21], the vessels in the skin, as well as the subcutaneous tissue become narrowed as a result of α2-adrenergic receptor stimulation [22], which is reflected in the reduction of skin temperature [23]. It has also been shown that vasoconstriction, caused by skin cooling, is the consequence of combining an increase in noradrenaline concentration and a decrease in NOS activity [24]. After the completion of the WBC treatments, the skin temperature becomes normalised very quickly [25]. Normal vasomotor function is particularly important in the inflamm-aging, which predisposes seniors to oxidative stress, increased vasoconstriction and CVD development [26]. Following the WBC procedures, an increase in NO concentration among the participants was shown [27], but so far, there are no studies regarding the influence of WBC on the activity of iNOS among seniors in connection with the potential generation of nitro-oxidative stress [28].

The aim of our research was to determine whether regularly repeated WBC treatments change the activity of iNOS and the concentration of its endogenous inhibitor—ADMA, as well as HCY and inflammatory markers. Furthermore, it was assessed whether WBC treatments cause nitro-oxidative stress in the older participants and if aerobic training modifies this answer.

The following hypotheses were formulated: (1) as a result of repeated exposure to cryogenic temperatures, the concentration of iNOS increases in seniors; (2) the increase in iNOS concentration following exposition to cryogenic temperatures is greater in endurance athletes; (3) repeated exposure to cryogenic temperatures does not induce nitro-oxidative stress.

## 2. Materials and Methods

The research was conducted in accordance with the Declaration of Helsinki and the research methodology was approved by the Bioethical Committee of the Regional Medical Chamber in Kraków (127/KBL/OIL/2013). The participants were informed in detail about the purpose and course of the study and about the possibility of withdrawing from participation in the project at any stage without giving justification. All of the subjects read the written information about the course of research, the required clothing and behaviour during the WBC procedures. The participants provided their written consent for voluntary participation in the trial.

### 2.1. Participants

Healthy, non-smoking Caucasian men aged 59 ± 6 years were enrolled in the study. Among the volunteers, there were 10 amateur athletes practicing in long-distance running—RUN (training 6.71 ± 5.79 years) and 10 untrained subjects—UTR.

Participants obtained medical qualifications for WBC procedures, and did not take any medications (e.g., antihypertensive, anti-inflammatory, hormonal, lipid-lowering etc.).

During the study period, participants did not use any wellness treatments (e.g., sauna, massage, hydrotherapy) other than WBC. Men who had participated in WBC procedures for treatment or stimulation purposes in the 6 months prior to inclusion in the project, were excluded from participation.

During the application of WBC treatments, the subjects did not change their physical activity (PA) or their current diet, they did not implement special diets (e.g., diabetic, low-calorie, vegetarian, vegan, etc.), supplements containing macro- and microelements, or vitamin supplements. The method of nutrition was assessed 3 times during the study (in weeks 1, 4 and 8) using the Dieta 5.0 computer program (Food and Nutrition Institute, Warsaw, Poland), based on the analysis of 7-day dietary diaries filled in daily by the participants [29]. On the basis of the 7-Day Physical Activity Recall [30], the participants’ PA levels were determined.

### 2.2. Somatic Measurements

Height (stadiometer Seca 217, Hamburg, Germany), lean body mass, fat mass and percentage of body fat were measured (Jawon IOI-353 Body Composition Analyzer, Gyeongsa, Korea). Body mass index (BMI) was calculated for each participant.

### 2.3. Whole-Body Cryotherapy Procedure

The participants underwent 24 WBC treatments in the Bamet KN-1 cryogenic chamber (Bamet, Wielka Wies, Poland), cooled with liquid nitrogen and in controlled conditions of temperature, air humidity and oxygen content at the level of 21–22% (2 independent EurOx oxygen probes. O2 G/E, Kraków, Poland). WBC treatments were performed 3 times a week (Monday, Wednesday and Friday) in the afternoon (2:00 p.m.–4:00 p.m.). Each treatment began with a 30-s stay in the vestibule at −60 °C, and then maintained in the temperature of approximately −130 °C for 3 min. During the procedure, the subjects moved slowly in a circle one after the other, without talking, without touching, changing the direction of their march every 30 seconds. The treatments were supervised by a physical therapist (camera, audio system). At any time, it was possible to terminate the procedure, both by the supervisor and the participant. During the procedure, participants were dressed in shorts, woollen high socks covering the knee joints, gloves, a band or cap covering the auricles, clogs, the nose and mouth covered with a surgical mask containing gauze. Before entering the cryochamber, the participants thoroughly dried the skin of sweat, and if applicable, removed their watches, jewellery and contact lenses.

### 2.4. Biochemical Analysis

For analysis, 30 min before the first WBC (T1) and 30 min after 12 WBC (T2) and 24 WBC (T3), and 7 days after the end of the 24 WBC series (T4), 6 mL of venous blood was collected from the antecubital vein into test tubes with the K2EDTA anticoagulant (dipotassium ethylenediaminetetraacetate dihydrate) (Becton Dickinson, Franklin Lakes, NJ, USA). After completing the WBC procedure, the participants did not consume any fluids or meals until blood was collected. Each time, blood was taken in a seated position after a 5-min rest. Immediately after collection, the blood was mixed by inverting the tube several times (avoiding shaking), and then immediately centrifuged for 15 min at 4 °C, RCF 1000 g (MPW-351R, Med. Instruments, Warsaw, Poland). Plasma was collected and stored until determinations at −70 °C (ULF 390 Arctiko, Esbjerg, Denmark).

The concentration of iNOS, 3-nitrotyrosine (3-NTR) and ADMA in the blood plasma was determined using the Human Nitric Oxide Synthase 2 SEA837Hu reagent kit, detection range 0–1000 pg/mL (Cloud-Clone Corp., Houston, TX, USA); Nitrotyrosine K7827 reagent kit, detection range 0–400 nmol/L (Immundiagnostik AG, Bensheim, Germany); ADMA K7860 reagent kit, detection range 0–2 µmol/L (Immundiagnostik AG, Bensheim, Germany). The concentration of interleukins IL-6, IL-1β and IL-10 was determined via the immunoenzymatic method (ELISA) method with the use of high-sensitivity reagent kits, respectively: Human IL-6 Immunoassay HS600B, detection range 0.156–10.0 pg/mL; Human IL-1β/IL-1F2 Immunoassay HSLB00C, detection range 0.125–8.0 pg/mL; Human IL-10 Immunoassay HS100C, detection range 0.78–50.0 pg/mL (R&D Systems, Inc., Minneapolis, MN, USA).

Plasma CRP concentration was determined via the immune-turbidimetric method using the Cardiac C-Reactive Protein (Latex) High Sensitive Test-CRPHS (Roche Diagnostics GmbH, Mannheim, Germany). The detection range of the CRPHS assay was 0.15–20 mg/L. The concentration of homocysteine in the blood plasma was determined by the enzymatic method using the Homocysteine Enzymatic Assay—HCYS Test (Roche Diagnostics GmbH, Mannheim, Germany). The detection range of the HCYS Assay was 3–50 µmol/L.

### 2.5. Statistical Analysis

The distribution of results for the analysed variables was checked via the Shapiro-Wilk test, while equality of variance was verified using the Levene test. For single measurements, the significance of differences between groups was assessed with the Student’s *t*-test for independent samples. Comparing the impact of WBC treatments on changes in the analysed variables, analysis of variance with repeated measures (ANOVA) was implemented, examining the influence of the main factors, i.e., physical activity (PA), whole-body cryotherapy (WBC) treatments, and PA × WBC interaction. The clinical effect size for ANOVA was calculated using partial eta squared (η^2^) and interpreted as 0.010–0.059 = low, 0.060–0.139 = moderate, ≥0.14 = high. Confidence intervals of the mean (95% CI) were determined for the changes in the level of individual variables after WBC. When the major factors were found to have significant influence, post-hoc analysis was performed using a planned comparison test. The statistical significance of the differences was set at a level of *p* < 0.05. The statistical power of the test (1-β) was also calculated. The STATISTICA 13.3 package (StatSoft, Inc., Tulsa, OK, USA) was used for the calculations.

## 3. Results

### 3.1. Participant Characteristics

#### 3.1.1. Somatic Characteristics and Medical Qualification

With comparable body mass (*p* > 0.05), the UTR group was characterised by significantly higher percentage of fat (*p* = 0.01) and BMI (*p* = 0.01) compared to RUN. Among the groups, there were no significant differences in the level of blood counts, in the level of fasting glucose or the content of glycated haemoglobin (*p* > 0.05). In terms of lipid profile components, only HDL concentration differed in both groups, being significantly higher in the RUN group (*p* = 0.03), but the concentration of fibrinogen was significantly higher in the UTR (*p* = 0.02) (Table 1).

#### 3.1.2. Level of Physical Activity

The men from the RUN group trained at a club in annual macrocycles according to the plans prepared by their coaches. They trained 3–5 times a week, the monthly training distance ranged from 55 to 150 km, they prepared for at least 2 marathons a year, also competed in at least 2 half-marathons, and 2–3 times a year, in a 10-km run. It was found that in the RUN group, the weekly duration of very hard (10 MET) and hard (6 MET) exercise was 4.06 ± 3.16 h and 5.33 ± 3.70 h, respectively. Men from the UTR group did not declare performing exercise of very hard intensity, while the hard exercise duration equalled 0.7 ± 1.25 h/week and was significantly shorter than in the RUN group (*p* < 0.01). The weekly duration of moderate intensity exercise (4 METs) was similar in both groups and totalled 6.17 ± 1.92 h in RUN and 4.18 ± 3.43 h in UTR (*p* > 0.05). The training men did not take part in sports competitions during the research period.

#### 3.1.3. Evaluation of Nutrition

The daily caloric content of the diet and the percentage of individual nutrients in the first week of the study were similar in the RUN and UTR groups (*p* > 0.05) and amounted to 2038.5 ± 629.5 kcal/day in the RUN group, 18.1 ± 2.2% for proteins, 48.2 ± 6.0% in the case of fats, 30.9 ± 5.7% for carbohydrates; in UTR, these values totalled 1672.2 ± 426.4 kcal/day, 19.7 ± 2.1% in the case of proteins, 46.5 ± 6.4% fats, and 32.9 ± 7.6% for carbohydrates. These values did not differ within (*p* > 0.05) or between groups (*p* > 0.05) at weeks 4 or 8 of WBC application.

### 3.2. Influence of Repeated Exposition to Cryogenic Temperatures on Level of Biochemical Indices

**Figure 1:**
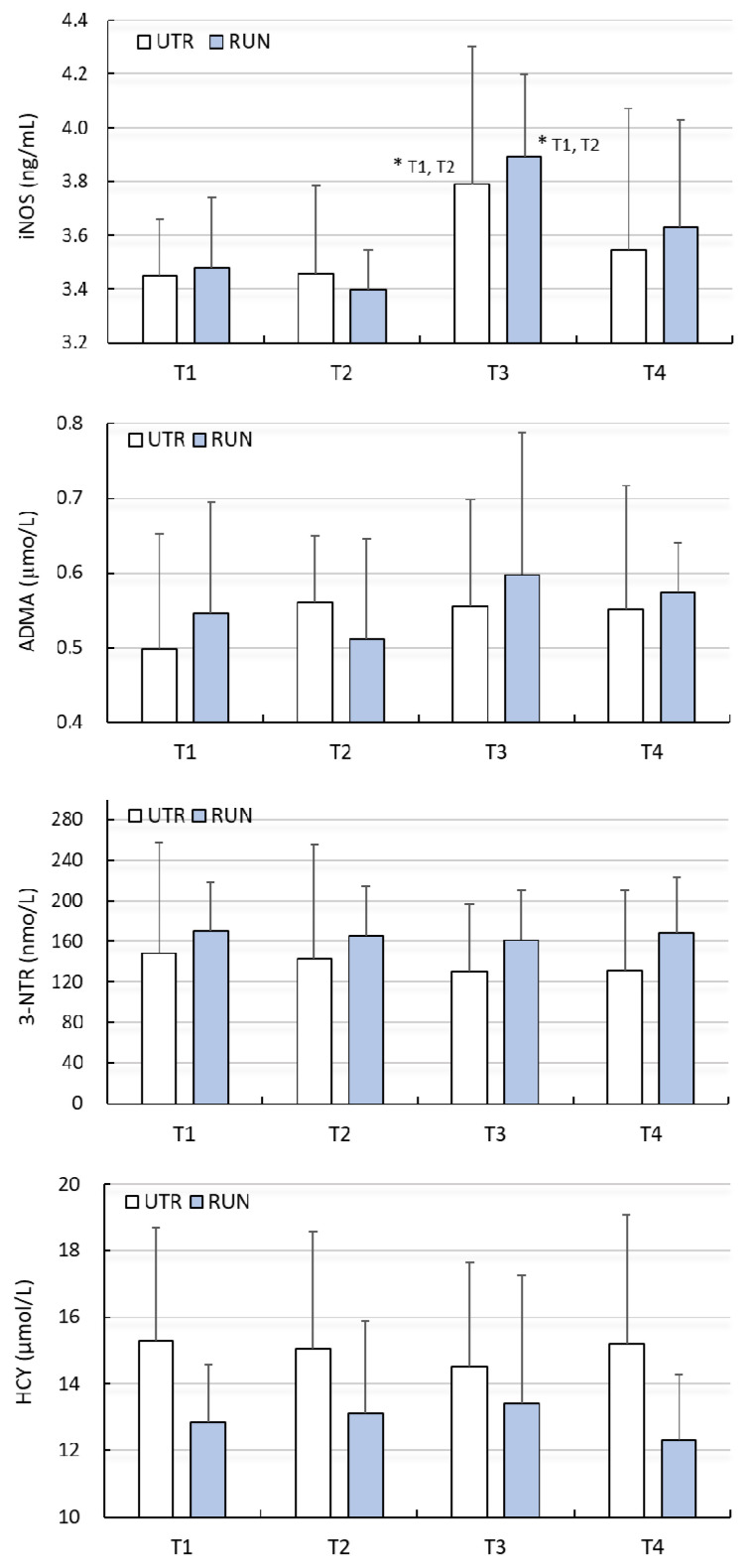
Concentration of inducible nitric oxide synthase (iNOS), asymmetric dimethylarginine (ADMA), 3-nitrotyrosine (3-NTR) and homocysteine (HCY) in blood plasma (mean ± standard deviation) in long-distance runners (RUN) and in untrained individuals (UTR) during the period of systemic cryotherapy (WBC) treatment and 7 days after its completion. *—statistically significant difference vs. T1 and T2 *(p* < 0.05); T1—measurement before 1 WBC, T2—measurement after 12 WBC, T3—measurement after 24 WBC, T4—measurement 7 days after completing WBC treatments.

A significant effect of WBC (large clinical effect size) on the concentration of iNOS was found (F = 5.95, *p* < 0.01), while there was no significant effect of PA on the concentration of iNOS (F = 0.16, *p* = 0.69). Changes in iNOS concentration under the influence of WBC in both groups were similar (ANOVA PA × WBC: F = 0.22, *p* = 0.88) (Table 2). Post-hoc analysis allowed to demonstrate that the concentration of iNOS, before beginning treatments (T1), was comparable (*p* > 0.05) in the RUN (3.48 ± 0.23 ng/mL) and UTR (3.45 ± 0.27 ng/mL) groups. In none of the groups did 12 WBC (T2) treatments change the iNOS concentration (*p* > 0.05). After 24 WBC (T3) applications, the concentration of iNOS in the RUN and UTR groups was significantly higher compared to T1 and T2, and amounted to 3.90 ± 0.54 ng/mL (*p* = 0.01) and 3.79 ± 0.32 ng/mL (*p* = 0.03), respectively (Figure 1). The change in iNOS concentration with regard to T3 compared to T1 in the RUN group was 0.42 ng/mL (95%CI 0.08–0.75; diff. 12%), while in the UTR group, its value totalled 0.34 ng/mL (95%CI 0.03–0.66; diff. 10%) (Table 2).

**Figure 2:**
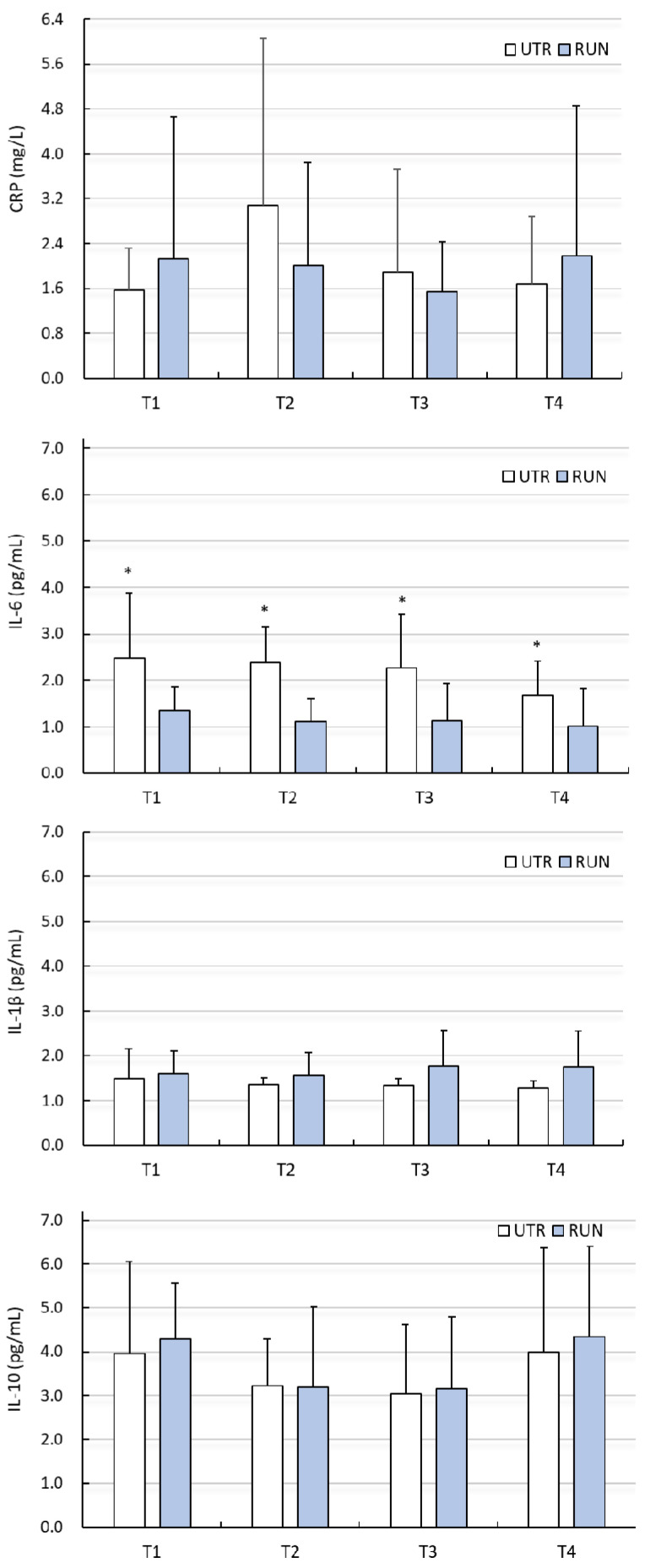
Concentration of C-reactive protein (CRP), interleukin 6 (IL-6), interleukin 1β (IL-1β), interleukin 10 (IL-10) in blood plasma (mean ± standard deviation) in long-distance runners (RUN) and in untrained individuals (UTR) during the period of systemic cryotherapy (WBC) treatment and 7 days after its completion. *—statistically significant difference vs. RUN *(p* < 0.05); T1—measurement before 1 WBC, T2—measurement after 12 WBC, T3—measurement after 24 WBC, T4—measurement 7 days after completing WBC treatments.

There were no significant differences (PA factor) between groups (except IL-1β, IL-6), or influence of WBC treatments with regard to changes in ADMA, 3-NTR, HCY, CRP, IL-1β or IL-10 levels (*p* > 0.05) (Figure 1 and Figure 2, Table 2 and Table 3). Despite the significant main effect (PA) for IL-1β (*p* = 0.03), post-hoc analysis indicated that differences between groups (RUN vs. UTR) in IL-1β concentrations concerned other comparisons than those in the analysed points (T1–T4). No effects of the interaction between PA × WBC on the levels of these biochemical indices were noted. The concentration of IL-6 at points T1–T4 was significantly higher in the UTR group compared to the RUN group (*p* < 0.05, large effect size), there was no significant effects of WBC on the level of IL-6 (*p* = 0.26), nor were there significant differences between groups in response to WBC (ANOVA PA × WBC: F = 0.70, *p* = 0.56) (Figure 2, Table 3).

## 4. Discussion

Our research is the first in which it has been demonstrated that the application of cryogenic temperatures increases the concentration of iNOS in the blood of senior men. This requires implementing a series of WBC treatments. The number of 12 WBC treatments is insufficient to achieve this effect. In our study, a significant increase in iNOS concentration occurred between the 12th and 24th WBC procedures and was similar in the group of men training long-distance running and those non-training.

Nitric oxide was formed as a result of iNOS activation, with a high concentration of the superoxide radical anion, which can form peroxynitrite and induce nitro-oxidative stress as well as inflammation. In previous studies, it has been indicated that older age and low physical activity result in higher levels of oxidative stress markers, which are associated with CVD risk factors, but no correlations have been found either between ox-LDL—an indicator of lipid oxidative damage—and NO, or between 3-NTR and NO levels in the blood [4]. In our research, there were no significant differences in the levels of iNOS and 3-NTR in the studied groups, despite differences in physical activity levels. Both groups responded with a similar increase in iNOS levels as a result of the WBC treatments. It is known that the increase in the bioavailability of vasodilating NO is mainly the effect of the eNOS isoform, and iNOS is induced by inflammation through the NF-kB pathway [31,32], but it has been found that under physiological conditions, due to physical training, iNOS is up-regulated, also having beneficial vasodilatory effects, as demonstrated in an animal model [33]. Animal models have also shown a reduction in aortic atherosclerosis with a simultaneous reduction in IL-6 and TNF-α expression and up-regulation of iNOS expression, and that inhibition of iNOS exacerbates atherosclerosis, reduces cortical perfusion and enhances ischemic neuronal damage and impairs Ach-dependent muscle relaxation of the smooth vessels [34]. The mechanism of these changes is not fully known. The damaging or protective effects of increased iNOS expression depend on the availability of the superoxide radical and the development of oxidative stress [34]. In our research, we did not find any changes in inflammatory markers as a result of WBC application. Moreover, no increases in the concentration of 3-NTR (an indicator of nitro-oxidative stress) were noted. Therefore, the increase of iNOS concentration in the blood obtained in our study, after completing the series of 24 WBC treatments, has beneficial effects, which may result in an increase in the bioavailability of nitric oxide. It has been previously shown that WBC increased NO levels while lowering IL-6 levels in the blood among patients with mild cognitive impairments (MCI), which was indicative of anti-inflammatory effects [27]. However, this was not confirmed in subsequent studies, in which no significant effect of WBC was demonstrated on the level of NO or inflammatory markers such as CRP, IL-6 and IL-10 among patients with depression [35] or those with MCI [36]. To date, there are no studies in which the activity of NOS isoforms as a result of WBC application would be assessed.

Stanek et al. [37] obtained the beneficial effects of WBC treatments for the endothelial function. As a result of 10 daily exposures in the cryochamber (3 min, −120 °C), followed by kinesiotherapy, an improvement in endothelial function was achieved in 40-year-old healthy men, manifested by a reduction in the level of inflammatory markers. There was a decrease in the concentration of sCD40L (soluble CD40 ligand), which is a platelet-derived pro-inflammatory mediator, a decrease in the concentration of amyloid A (acute phase protein of inflammation) and a decrease in MPO (myeloperoxidase) activity [37]. Anti-inflammatory effects of exposure to cryogenic temperatures have also been demonstrated in obese men [38,39].

IL-6 is a multifunctional cytokine that can play pro- and anti-inflammatory roles [40]. Physical exercise not causing microdamage increases the concentration of IL-6, but does not increase the concentration of other pro-inflammatory cytokines [41]. In such a situation, IL-6 has a metabolic effect that stimulates lipolysis and lipid oxidation [41]. Diseases cause a significant increase in the concentration of IL-6 in the blood, with a simultaneous increase in the concentration of other pro-inflammatory cytokines, such as TNF-α and IL-1β. With age, the level of IL-6 in the blood increases [42], which may indicate chronic, low-grade inflammation [26], while regular physical activity reduces the concentration of IL-6 in the blood in seniors [43]. This was reflected in our research, because untrained men had a higher concentration of IL-6 compared to the runners. The different levels of physical activity of our subjects did not have a significant effect on the level of other inflammatory markers such as CRP, IL-1β or IL-10, but both in the RUN and UTR groups, there were large differences within these markers.

The use of daily, repeated WBC treatments caused increases in IL-6 [44,45,46,47], IL-10 [45,48,49], IL-1ra [50] and a decrease in IL-1α [45], IL-1β [49,50,51], IL-2, IL-8 [48] and TNF-α [46,49], which indicates the anti-inflammatory effect of WBC treatments. Contrary to these results, we did not find significant changes in the level of inflammatory markers. The concentration of acute phase inflammatory protein—CRP, IL-6 and IL-1β did not change significantly in any of the groups, and no changes in the anti-inflammatory IL-10 concentration were found. Also, according to other studies, the use of WBC treatments has no effect on the concentration of IL-1β, IL-12 and TNF-α [45] or IL-6 and IL-10 [50]. Perhaps the lack of anti-inflammatory WBC effects in our research is due to our use of treatments every other day and/or too few exposures.

Furthermore, we did not achieve a significant reduction in the concentration of ADMA and HCY, which was the expected effect from the use of WBC. However, when analysing the individual results prior to WBC, according to the clinical norms [11], we found mild hyperhomocysteinemia (HCY 12.0–30.0 µmol/L) in 16 individuals, including 7 from the RUN group. After the completion of the 24 WBC series, the lower limit of the HCY concentration range observed in the subjects underwent a decrease, and mild hyperhomocysteinemia was observed in 12 subjects. Positive individual effects were also obtained in the concentration of CRP. Before initiating WBC procedures, 2 people from the RUN group and 8 subjects from the UTR group were at an average risk of CVD (CRP 1.0–3.0 mg/L), while according to Buckley et al. [52], a high risk of developing CVD was found in 2 people from the RUN group and 1 person from the UTR group (CRP > 3.0 mg/L). After completing the series of 24 WBC procedures, an average risk of CVD was reported in 9 people, and high in 2 of them.

iNOS activation may be the reason for the intensification of nitro-oxidative stress [34], although WBC treatments show an antioxidant effect [17,18,19]. Increasing the antioxidant defence as a result of WBC may reduce the level of the superoxide radical anion and the protective effects of increasing the bioavailability of NO as a result of the increased concentration of iNOS found after the treatments. A reduction in oxidative stress has been shown in both healthy individuals [19,37] and patients with ankylosing spondylitis [18] undergoing systemic cryotherapy followed by kinesiotherapy. After 10 WBC treatments combined with exercise, a significant reduction in total oxidative status and the oxidative stress index was observed in both groups, as well as a significant increase in the total antioxidant capacity of the blood [18,19]. In healthy men, an increase in the activity of superoxide dismutase in plasma and erythrocytes was also found [19], as well as a decrease in the activity of paraoxonase-1 [37]. Also, as a result of the influence of cryogenic temperatures alone, beneficial changes were found to improve the systemic redox status [53,54]. The effect of 10 WBC treatments, which were performed daily in young, healthy men, was a significant increase in CAT activity in erythrocytes, while an increase in the number of treatments to 20 resulted in a significant rise in SOD activity [54]. We obtained a similar effect in our previous research [55], in which, after the application of a 12 WBC treatment series, applied every other day, there was a significant increase in SOD activity among young men in training, and increasing the number of treatments to 24 enhanced this effect and, at the same time, increased SOD activity in the group of non-training men. After a series of 24 WBC blood treatments in the group of senior long-distance runners, we found a significant increase in the concentration of reduced glutathione (GSH)—antioxidant [56]. In the present study, we assessed nitro-oxidative stress in senior males by determining the concentration of 3-NTR as a result of 12 and 24 WBC treatments. We found no changes in the concentration of this marker. We did not note any effects of WBC on the level of protein nitration. Furthermore, no correlation was found between the concentration of iNOS and 3-NTR, which proves the lack of intensification in nitro-oxidative stress as a result of iNOS up-regulation. We have shown that systematic, repeated (every-other-day) exposure to the effects of cryogenic temperatures, in the amount of 24 treatments-exposure, has an effect on endurance-training and non-training elderly men, based on up-regulation of iNOS in the blood, which may indicate the beneficial influence of cryogenic temperatures on the bioavailability of nitric oxide and vascular vasodilatation in seniors, because no nitro-oxidative stress or inflammation occurred. Nonetheless, our research has limitations due to the lack of direct assessment of changes in nitric oxide levels and eNOS activity as a result of WBC activity. A clear conclusion regarding the increase in NO bioavailability and the anti-atherosclerotic function of WBC treatments in seniors requires further examination.

## 5. Conclusions

As a result of applying 24 WBC treatments, using the every-other-day model, iNOS concentration increased in older men, regardless of their physical activity level and without changes in nitro-oxidative stress or inflammation markers levels.

## Figures and Tables

**Table 1:** Somatic characteristics and medical qualification of study participants (mean ± SD).

Variables	Total	RUN	UTR	*p* Value
Body height (cm)	171.95 ± 7.87	175.20 ± 6.37	168.70 ± 8.17	0.06
Body mass (kg)	76.96 ± 7.16	76.33 ± 6.32	77.59 ± 8.21	0.70
Total body fat (%)	23.87 ± 5.35	21.33 ± 4.36	26.40 ± 5.20	0.01
Body mass index (kg/m^2^)	26.08 ± 2.20	24.87 ± 1.28	27.28 ± 2.32	0.01
Erythrocytes (10^6^/µL)	4.84 ± 0.48	4.75 ± 0.55	4.93 ± 0.41	0.42
Haemoglobin (g/dL)	14.85 ± 1.20	14.52 ± 1.17	15.18 ± 1.19	0.23
Haematocrit (%)	43.36 ± 3.21	42.47 ± 3.02	44.25 ± 3.30	0.22
ESR (mm/h)	8.11 ± 7.56	6.70 ± 6.52	9.67 ± 8.69	0.41
Leucocytes (10^3^/µL)	6.48 ± 1.49	6.31 ± 1.53	6.65 ± 1.51	0.62
Platelets (10^3^/µL)	236.55 ± 53.73	242.10 ± 40.60	231.00 ± 66.16	0.29
Fasting glucose (mg/dL)	96.65 ± 9.07	93.31 ± 6.96	100.36 ± 10.06	0.09
Glycated haemoglobin (%)	5.30 ± 0.33	5.43 ± 0.13	5.17 ± 0.42	0.18
TC (mg/dL)	201.06 ± 39.29	206.15 ± 38.82	195.41 ± 41.43	0.57
HDL (mg/dL)	63.09 ± 16.94	70.73 ± 11.38	54.61 ± 18.60	0.03
LDL (mg/dL)	121.53 ± 34.38	119.45 ± 34.58	123.83 ± 36.09	0.79
TG (mg/dL)	82.98 ± 28.89	80.69 ± 28.50	85.52 ± 30.83	0.68
AIP	0.11 ± 0.22	0.04 ± 0.17	0.20 ± 0.24	0.11
Total protein (g/L)	71.57 ± 3.39	71.68 ± 3.98	71.45 ± 2.88	0.88
Fibrinogen (g/L)	2.71 ± 0.54	2.46 ± 0.57	2.97 ± 0.37	0.02

*p* < 0.05—significant differences active vs. inactive group; RUN: long distance runners, UTR: untrained—control group, ESR: erythrocyte sedimentation rate, TC: total cholesterol, HDL: high density lipoproteins, LDL: low density lipoproteins, TG: triglycerides, AIP: atherogenic index of plasma = log(TG/HDL).

**Table 2:** Changes in nitro-oxidative stress indices concentration in the period of whole-body cryotherapy (WBC) application in the group of long-distance runners (RUN) and in the group of non-training men (UTR).

								ANOVA	
Variable	WBC		Total	RUN	UTR		PA	WBC	PA × WBC
iNOS	T1	Mean ± SD	3.46 ± 0.25	3.48 ± 0.23	3.45 ± 0.27	F	0.16	5.95	0.22
(ng/mL)	Δ_T2vsT1_	Mean (95% CI)	−0.04 (−0.18, 0.11)	−0.08 (−0.30, 0.14)	0.01 (−0.22, 0.24)	p	0.69	<0.01	0.88
	Δ_T3vsT1_	Mean (95% CI)	0.38 (0.17, 0.59)	0.42 (0.08, 0.75)	0.34 (0.03, 0.66)	η^2^	0.01	0.25	0.01
	Δ_T4vsT1_	Mean (95% CI)	0.13 (−0.15, 0.40)	0.15 (−0.23, 0.54)	−0.39 (0.59, −0.70)	1-β	0.07	0.94	0.09
ADMA	T1	Mean ± SD	0.52 ± 0.16	0.55 ± 0.16	0.50 ± 0.16	F	0.08	1.25	1.01
(µmol/L)	Δ_T2vsT1_	Mean (95% CI)	0.01 (−0.05, 0.08)	−0.04 (−0.10, 0.03)	0.06 (−0.05, 0.18)	p	0.78	0.30	0.40
	Δ_T3vsT1_	Mean (95% CI)	0.05 (−0.01, 0.12)	0.05 (−0.04, 0.15)	0.06 (−0.06, 0.18)	η^2^	<0.01	0.06	0.05
	Δ_T4vsT1_	Mean (95% CI)	0.04 (−0.03, 0.11)	0.03 (−0.06, 0.12)	0.05 (−0.07, 0.17)	1-β	0.06	0.31	0.26
3-NTR	T1	Mean ± SD	159.30 ± 86.86	170.40 ± 50.29	148.20 ± 114.57	F	0.68	1.13	0.43
(nmol/L)	Δ_T2vsT1_	Mean (95% CI)	−5.20 (−15.70, 5.30)	−5.20(−26.53, 16.13)	−5.20 (−14.65, 4.25)	p	0.42	0.35	0.73
	Δ_T3vsT1_	Mean (95% CI)	−13.70 (−31.78, 4.38)	−9.20 (−30.95, 12.55)	−18.20 (−51.60, 15.20)	η^2^	0.04	0.06	0.02
	Δ_T4vsT1_	Mean (95% CI)	−9.40 (−29.13, 10.33)	−2.00 (−34.04, 30.04)	−16.80 (−45.63, 12.03)	1-β	0.12	0.29	0.13
HCY	T1	Mean ± SD	14.07 ± 3.04	12.84 ± 1.84	15.30 ± 3.57	F	2.75	0.11	0.75
(µmol/L)	Δ_T2vsT1_	Mean (95% CI)	0.02 (−1.22, 1.25)	0.28 (−1.09, 1.65)	−0.25 (−2.6, 2.12)	p	0.11	0.951	0.528
	Δ_T3vsT1_	Mean (95% CI)	−0.10 (−1.62, 1.42)	0.57 (−1.37, 2.51)	−0.77 (−3.42, 1.88)	η^2^	0.13	0.01	0.04
	Δ_T4vsT1_	Mean (95% CI)	−0.30 (−1.88, 1.27)	−0.51 (−1.56, 0.54)	−0.10 (−3.42, 3.22)	1-β	0.35	0.07	0.20

SD: standard deviation, CI: confidence interval, T1: measurement before 1 WBC, T2: measurement after 12 WBC, T3: measurement after 24 WBC, T4: measurement 7 days after completing WBC treatments, Δ_vsT1_: difference in results for points T2, T3 and T4 vs. T1; iNOS: inducible nitric oxide synthase, ADMA: asymmetric dimethylarginine, 3-NTR: 3- nitrotyrosine, HCY: homocysteine; η^2^: partial eta squared (0.010–0.059 = small clinical effect, 0.060–0.139 = moderate clinical effect, ≥ 0.14 = large clinical effect), 1-β: power of statistical test.

**Table 3:** Changes in inflammation indices concentration during the period of whole-body cryotherapy (WBC) treatment in the group of long-distance runners (RUN) and in the group of untrained men (UTR).

								ANOVA	
Variable	WBC		Total	RUN	UTR		PA	WBC	PA × WBC
CRP	T1	Mean ± SD	1.84 ± 1.93	2.12 ± 2.66	1.57 ± 0.79	F	0.02	0.83	0.94
(mg/L)	Δ_T2vsT1_	Mean (95% CI)	0.70 (−0.74, 2.13)	−0.12 (−2.23, 1.99)	1.51 (−0.70, 3.72)	p	0.89	0.48	0.43
	Δ_T3vsT1_	Mean (95% CI)	−1.23 (0.97, 2.36)	−0.59 (−2.53, 1.36)	0.33 (−1.07, 1.73)	η^2^	<0.01	0.04	0.05
	Δ_T4vsT1_	Mean (95% CI)	−0.93 (1.11, 2.18)	0.06 (−2.01, 2.14)	0.12 (−0.79, 1.02)	1-β	0.05	0.22	0.24
IL-6	T1	Mean ± SD	1.76 ± 1.05	1.34 ± 0.98	2.18 ± 0.98 *^,^^RUN^	F	17.72	1.39	0.70
(pg/mL)	Δ_T2vsT1_	Mean (95% CI)	−0.02 (−0.52, 0.49)	−0.23 (−0.83, 0.37)	0.19 (−0.72, 1.11)	p	<0.01	0.26	0.56
	Δ_T3vsT1_	Mean (95% CI)	−0.06 (−0.60, 0.49)	−0.20 (−0.88, 0.47)	0.09 (0.90, 1.08)	η^2^	0.50	0.07	0.04
	Δ_T4vsT1_	Mean (95% CI)	−0.41 (−0.94, 0.12)	−0.32 (−1.00, 0.36)	−0.51 (−1.47, 0.45)	1-β	0.98	0.35	0.19
IL-1β	T1	Mean ± SD	1.55 ± 0.62	1.60 ± 0.54	1.49 ± 0.71	F	5.39	0.10	0.46
(pg/mL)	Δ_T2vsT1_	Mean (95% CI)	−0.08 (−0.45, 0.28)	−0.03 (−0.62, 0.96)	−0.13 (−0.68, 0.42)	p	0.03	0.96	0.71
	Δ_T3vsT1_	Mean (95% CI)	0.01 (−0.43, 0.45)	0.17 (−0.62, 0.96)	−0.15 (−0.69, 0.39)	η^2^	0.23	0.01	0.03
	Δ_T4vsT1_	Mean (95% CI)	−0.02 (−0.46, 0.42)	0.16 (−0.67, 0.98)	−0.20 (−0.69, 0.30)	1-β	0.59	0.07	0.14
IL-10	T1	Mean ± SD	4.13 ± 1.79	4.30 ± 1.34	3.96 ± 2.21	F	0.13	2.24	0.06
(pg/mL)	Δ_T2vsT1_	Mean (95% CI)	−0.92 (−1.95, 0.12)	−1.11 (−2.75, 0.54)	−0.73 (−2.32, 0.87)	p	0.72	0.09	0.98
	Δ_T3vsT1_	Mean (95% CI)	−1.02 (−2.14, 0.10)	−1.14 (−2.64, 0.36)	−0.90 (−2.88, 1.08)	η^2^	0.01	0.11	<0.01
	Δ_T4vsT1_	Mean (95% CI)	0.04 (−1.56, 1.64)	0.04 (−1.87, 1.96)	0.04 (−2.96, 3.03)	1-β	0.06	0.54	0.06

SD: standard deviation, CI: confidence interval, T1: measurement before 1 WBC, T2: measurement after 12 WBC, T3: measurement after 24 WBC, T4: measurement 7 days after completing WBC treatments, Δ_vsT1_: difference in results for points T2, T3 and T4 vs. T1; CRP: C-reactive protein, IL-6: interleukin 6, IL-1β: interleukin 1β, IL-10: interleukin 10; * statistically significant difference (*p* < 0.05), η^2^: partial eta squared (0.010–0.059 = small clinical effect, 0.060–0.139 = moderate clinical effect, ≥ 0.14 = large clinical effect), 1-β: power of statistical test.

## Data Availability

Raw data will be available on reasonable request from the corresponding author within the rules of data privacy protection and ethical approval.
